# An exploratory study of unexplained concentration of ^18^F-PSMA-1007 in the bladder for prostate cancer PET/CT imaging

**DOI:** 10.3389/fmed.2023.1238333

**Published:** 2023-08-31

**Authors:** Jun Dang, Yutang Yao, Yingchun Li, Xiaofei Tan, Zhenyan Ye, Yi Zhao, Shiwei Qing, Ying Kou, Xiao Jiang, Hao Lu, Shirong Chen, Meng Zhao, Zhuzhong Cheng

**Affiliations:** ^1^Department of Nuclear Medicine, Sichuan Clinical Research Center for Cancer, Sichuan Cancer Hospital and Institute, Sichuan Cancer Center, Affiliated Cancer Hospital of University of Electronic Science and Technology of China, Chengdu, China; ^2^Department of Nuclear Medicine and Radiotherapy, Air Force Hospital of Western Theater Command, Chengdu, China

**Keywords:** prostate cancer, ^18^F-PSMA-1007, PET, bladder uptake, positron emission tomography

## Abstract

^18^F-PSMA-1007 PET/CT imaging is increasingly used for the diagnosis, staging, and efficacy assessment of patients with prostate cancer. Compared with other PSMA tracers, ^18^F-PSMA-1007 is mainly cleared by the liver and bile and has lower urinary clearance, thus allowing a better assessment of the lesions around the bladder. However, there were some patients who showed an obvious concentration of the ^18^F-PSMA-1007 in the bladder, which may affect the observation of peripheral lesions, but the mechanism of this change is unknown. The aim of this study was to explore the cause of bladder ^18^F-PSMA-1007 concentration by assessing the clinical and imaging characteristics of ^18^F-PSMA-1007 PET/CT scans. A total of 284 patients were included in this retrospective study, and their clinical characteristics such as age, height, weight, Gleason score, metastases, different treatment methods, the level of liver and kidney function, PSA level, and imaging characteristics such as ^18^F-PSMA-1007 injected activity, the interval between injection to scan, physiological distribution (parotid gland, kidney, liver, spleen, intestine, obturator internus), pathological distribution (prostate lesions, metastases) were collected, and were compared after subgrouping using bladder urine SUV_max_. This study showed that the distribution of bladder ^18^F-PSMA-1007 was not correlated with the above clinical and imaging characteristics, so further studies are needed to find the explanations, and thus to improve the disease assessment of this type of prostate cancer patients.

## 1. Introduction

Prostate cancer is the second most common cancer among the world’s males and one of the leading causes of death in men (1), with the average age of newly diagnosed prostate cancer being approximately 66 years (2). Thus prostate cancer remains an important health problem.

Accurate staging of prostate cancer (PCa) is critical to the management of the disease and influences how patients are treated. Magnetic resonance imaging (MRI) has become the primary imaging modality for primary detection and localization of PCa. Bone scintigraphy and conventional abdominal imaging are recommended for the staging of intermediate and high-risk PCa, but they have been increasingly replaced by new types of imaging (3, 4). PSMA PET/CT is considered a valuable imaging method and has become the preferred staging modality for prostate cancer because of its excellent sensitivity and specificity (5, 6). PSMA is a transmembrane glycoprotein, overexpressed in prostate cancer cells, and radiolabeled small molecules with high affinity to its active extracellular centers underlie the mechanism of this imaging technique (7). Commonly used tracers for PET/CT evaluation of patients with prostate cancer include ^68^Ga-PSMA-11, ^18^F-DCFPyL, and ^18^F-PSMA-1007 (5, 6, 8, 9).

Noticeably, ^68^Ga-PSMA-11 and ^18^F-DCFPyL are mainly cleared through the kidney and caused a large amount of tracer accumulation in the bladder (10, 11), which can interfere with the detection of some lesions in the prostate and the pelvic region, leading to confusion between lesions adjacent to the bladder and the distribution of the tracers in the bladder. Compared to other ^18^F or ^68^Ga-labeled PSMA-targeted tracers, ^18^F-PSMA-1007 is mainly cleared by the liver and bile, temporarily retained in the renal parenchyma, and has a lower urinary clearance rate. The substantial reduction of tracers in the bladder allows thus well-evaluated lesions in the prostate and the pelvic region. Unexpectedly, an obvious concentration of ^18^F-PSMA-1007 can be observed in the bladder of some patients, however, which will affect the detection of lesions in the prostate and adjacent areas. This is especially in patients with clinical suspicion of biochemical recurrence, as long-term androgen deprivation therapy (ADT) will significantly reduce the visibility of prostate lesions on PSMA PET/CT (12). As the cause of this change in urine during ^18^F-PSMA-1007 metabolism is unknown, we propose to explore the cause of the high uptake of ^18^F-PSMA-1007 in bladder urine by assessing the clinical characteristics and imaging characteristics of the patient.

## 2. Materials and methods

### 2.1. Patients

Retrospective analysis of patients who underwent ^18^F-PSMA-1007 PET/CT imaging at our hospital from November 2018 to March 2023. The clinical data and imaging data were collected, including age, height, weight, treatment methods, histopathology, metastasis, liver and kidney function indicators 1 month before and after ^18^F-PSMA-1007 PET/CT imaging [albumin, globulin, alanine transaminase (ALT), aspartate transaminase (AST), total bilirubin, glomerular filtration rate (GFR), creatinine, uric acid] and TPSA, ^18^F-PSMA-1007 injected activity, the interval between drug injection and scan start, standardized uptake values (SUV) for normal organs (parotid gland, kidney, liver, spleen, intestine, obturator internus, bladder) and prostate lesions, and metastases. The study was ethically approved by the Institutional Ethics Committee (Ethics Committee of Sichuan Cancer Hospital, JS-2017-01-02). All patients signed a written informed consent form.

### 2.2. Radiosynthesis and quality control

^18^F-PSMA-1007 was produced in an automated radiosynthesizer (Sumitomo, Japan) according to the previously described one-step method (13). PSMA-1007 precursor dimethyl sulfoxide (DSMO) was obtained from ABX (Advanced Biochemical Compound GmbH, Radeberg, Germany). PSMA-1007 precursor (2 mg, 1.2 mL) dissolved in anhydrous DSMO was then added to the reactor and radiolabeled at 85°C for 10 min, and the liquid was then loaded onto PS-H + and C18ec. The final product was eluted with 4 mL of 30% ethanol and sterile filtered through a 0.22 μm filter (Millipore, MA). Radiochemical purity was tested using high-performance liquid chromatography (HPLC, Shimadzu, Japan) and thin-layer chromatography (TLC, Eckert & Ziegler, MA). Final product quality control includes appearance, color, clarity and radionuclide purity and meets acceptance criteria.

### 2.3. Imaging procedures

The patients were not specially prepped on the day of ^18^F-PSMA-1007 PET/CT scanning. A Biograph mCT-64 scanner (Siemens, Germany) was used. The scanning was performed about 180 min (14) after intravenous injection of ^18^F-PSMA-1007. The localizer was positioned with a scout head view. Low-dose CT (120 kV/110 mA) was then performed for anatomical localization and attenuation correction. Single-bed emission scans were obtained in three-dimensional mode (acquisition time, 3 min). Reconstruction of data was done using an ordered subset expectation maximization iterative reconstruction algorithm (three iterations, 21 subsets). The emission data were corrected for random, scatter, and decay.

### 2.4. Image analysis

Two nuclear medicine physicians who were experienced in PET/CT image assessment evaluated the PET/CT images. All measurements were completed in low-dose images, SUV_max_ and SUV_mean_ of prostate lesions and metastases were measured and recorded using the ROI technique, while maximum standardized uptake value (SUV_max_) and mean standardized uptake value (SUV_mean_) of parotid gland, kidney, liver, spleen, intestine, obturator internus and bladder were measured, avoiding areas where any lesions were present. Based on previous studies, the obturator internus was selected as the background (15, 16). Prostate lesions were defined as when tracer uptake was focal and higher than the surrounding prostate tissue (16). Metastatic foci are defined as when there are significant morphological changes in other soft tissues and bone, while the corresponding area shows higher than normal radiotracer uptake (17). According to Vollnberg et al. (18), we excluded regions of increased ^18^F-PSMA-1007 in the absence of morphological changes. Benign lesions are identified based on typical pitfalls in PSMA ligand PET imaging (e.g., ganglia, fractures, degenerative changes, and nonspecific lymph nodes) and CT information (19). Then the SUV_max_ =5 (14) was chosen as the criterion to divide the patients into a low uptake group and an increased uptake group. Based on our observations in clinical practice and the characteristics of the population distribution, the group was further divided into a moderate uptake group (5 <SUV_max_ ≤7.5) and a high uptake group (SUV_max_ >7.5).

### 2.5. Statistical analyses

Statistical analysis was performed using SPSS software version 26.0 (IBM Corporation). The measurement data were presented as mean ± standard deviation and numeration data were presented as *n* (%). The chi-square or Fisher exact test was used for categorical variables, and the Mann–Whitney *U* and Kruskal–Wallis tests were used for continuous variables. *p* < 0.05 was considered significant.

## 3. Results

### 3.1. Baseline demographics

**Figure 1:**
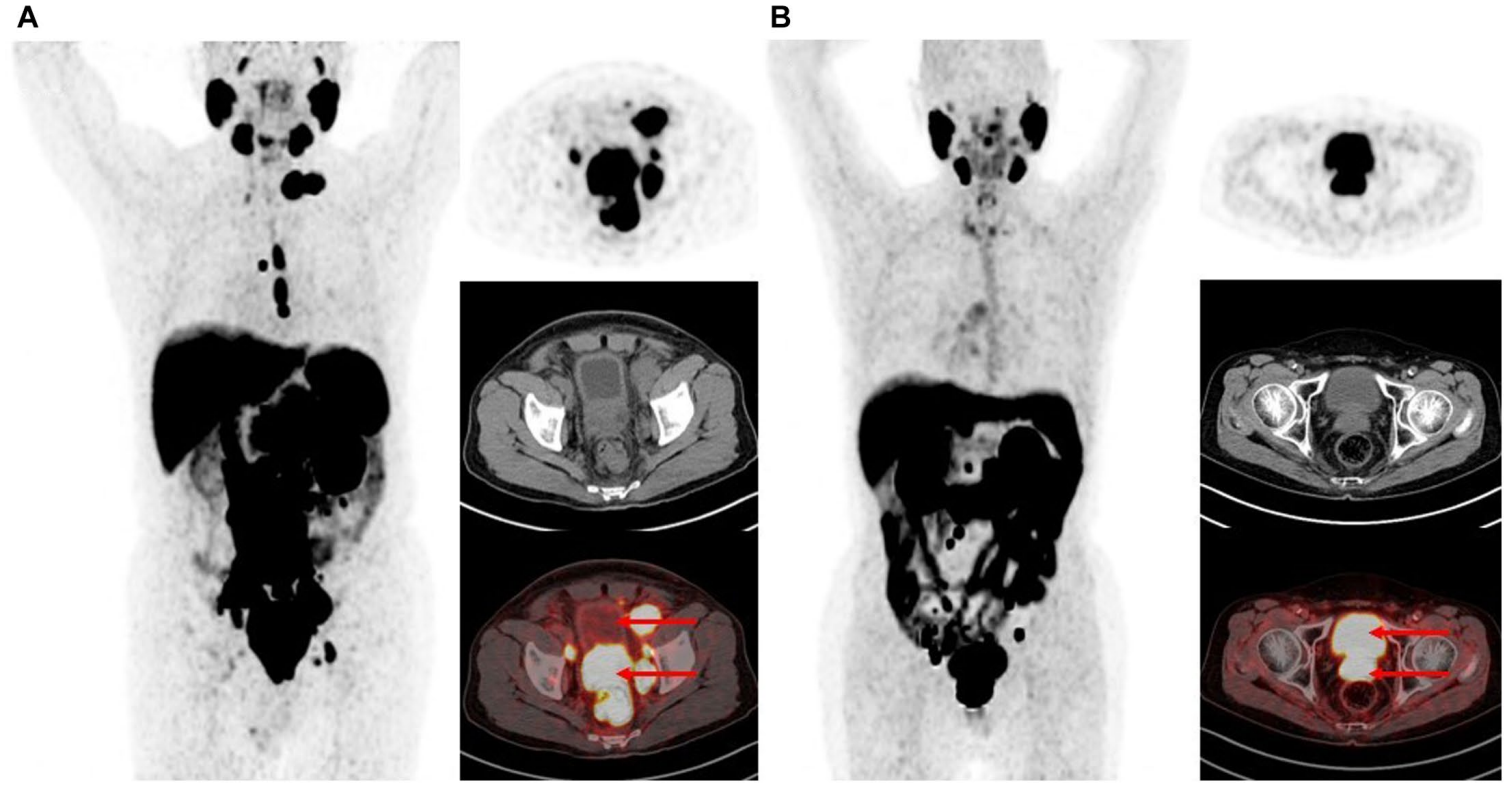
Maximum intensity projections and PET, CT, fusion image of the urinary bladder uptake on ^18^F-PSMA-1007 PET/CT. **(A)** Bladder SUV_max_ is 1.8. **(B)** Bladder SUV_max_ is 17.0.

A total of 263 patients underwent 284 ^18^F-PSMA-1007 PET/CT imaging (Table 1). The mean age at the time of the ^18^F-PSMA-1007 PET/CT scan was 68.4 ± 8.2 years. 32.7% (93/284) patients performing PET-CT were for staging. 67.3% (191/284) patients performing PET-CT were for restaging after treatment, of whom 22.2% (63/284) received androgen deprivation therapy (with or without chemotherapy), 23.6% (67/284) received radical prostatectomy only, 16.5% (47/284) received surgery combined with other treatments, 2.8% (8/284) received radiotherapy (with or without chemotherapy), 1.1% (3/284) received chemotherapy alone, and 1.1% (3/284) received targeted therapy. The mean TPSA was 99.2 ± 385.2. The mean time from injection of ^18^F-PSMA-1007 to the start of the scan was 182.8 ± 20.2 min. ^18^F-PSMA-1007 injected activity was 8.8 ± 1.1 mCi. One hundred seventy five patients had detectable prostate lesions on 181 scans and SUV_max_ was 25.4 ± 550.9 (range 4.1–175.1). 166 patients had detectable metastatic lesions on 182 scans and SUV_max_ was 22.5 ± 610.1 (range 1.9–216.1). The SUV_max_ of the bladder was 3.6 ± 10.5 with a range of 0.4–21.7. As shown in Figure 1, patients presented with various degrees of bladder tracer uptake, sometimes very high.

**Table 1:** Characteristics of the total patients.

Parameters	Values
Number of patients	263 (100%)
Number of scans	284 (100%)
Age (y)	68.4 ± 8.2
Height (cm) (*n* = 200)	165.6 ± 7.1
Weight (kg)	66.9 ± 9.7
TPSA (ng/mL) (*n* = 202)	99.2 ± 385.2
*Gleason score (n = 143)*	
6	7 (4.9%)
7	39 (27.3%)
8	36 (25.2%)
9	49 (34.2%)
10	12 (8.4%)
*Metastases*	
Yes	182 (64.1%)
No	102 (35.9%)
*Type of therapy*	
Initial diagnosis	93 (32.7%)
Radical prostatectomy	67 (23.6%)
ADT (with or without chemotherapy)	63 (22.2%)
Radical prostatectomy with other treatments	47 (16.5%)
Radiotherapy (with or without chemotherapy)	8 (2.8%)
Chemotherapy	3 (1.1%)
Targeted therapy	3 (1.1%)
^18^F-PSMA-1007 injected activity (mCi)	8.8 ± 1.1
Injection-to-scan interval (min)	182.8 ± 20.2
Bladder SUV_max_	3.6 ± 3.2
Prostate SUV_max_ (*n* = 181)	25.4 ± 23.5
Metastases SUV_max_ (*n* = 182)	22.5 ± 24.7
Parotid gland SUV_max_ (*n* = 282)	27.6 ± 8.5
Liver SUV_max_	14.0 ± 3.8
Kidney SUV_max_	29.2 ± 8.4
Spleen SUV_max_ (*n* = 283)	14.8 ± 4.9
Intestine SUV_max_	12.4 ± 5.0
Obturator internus SUV_max_	0.7 ± 0.2

TPSA, total prostate-specific antigen; ADT, androgen deprivation therapy; PSMA, prostate-specific membrane antigen; SUV_max_, maximum standardized uptake value; SUV_mean_, mean standardized uptake value.

### 3.2. Clinical characteristics

The clinical characteristics of patients with high (SUV_max_ >7.5), moderate (5 <SUV_max_ ≤7.5) and low uptake (SUV_max_ ≤5) of the bladder on ^18^F-PSMA-1007 PET/CT are shown in Table 2.23.6% of patients had SUV_max_ >5 and 10.6% had SUV_max_ >7.5 of the bladder. The Patient’s age, height, weight, albumin, globulin, ALT, AST, total bilirubin, creatinine, uric acid, GFR, TPSA, gleason score, metastasis, and different treatment methods were not related to the distribution of ^18^F-PSMA-1007 in the bladder (*p* > 0.05).

**Table 2:** Clinical characteristics stratified by SUV_max_ of the bladder.

Clinical characteristics	SUV_max_ ≤5	SUV_max_ 5–7.5	SUV_max_ >7.5	*p*
Number of scans, *n* (%)	217 (76.4%)	37 (13%)	30 (10.6%)	
Age (y)	68.1 ± 8.4	69.5 ± 7.8	68.7 ± 7.2	0.694
Height (cm) (*n* = 200)	165.8 ± 7.2	164.4 ± 7.6	166.4 ± 5.9	0.622
Weight (kg)	66.5 ± 9.5	67.5 ± 10.5	68.9 ± 9.6	0.414
*Blood parameters*				
TPSA (ng/mL) (*n* = 202)	100.6 ± 410.8	117.7 ± 387.2	71.8 ± 152.3	0.398
Albumin (g/L) (*n* = 122)	40.6 ± 4.5	39.4 ± 3.8	40.2 ± 3.9	0.617
Globulin (g/L) (*n* = 122)	25.9 ± 4.1	24.1 ± 4.1	25.6 ± 4.2	0.352
ALT (U/L) (*n* = 122)	25.5 ± 15.7	20.0 ± 14.5	21.4 ± 13.6	0.166
AST (U/L) (*n* = 122)	23.8 ± 9.3	26.1 ± 19.0	20.4 ± 32.9	0.392
Total bilirubin (μmol/L) (*n* = 122)	12.0 ± 6.7	9.2 ± 4.3	12.2 ± 6.0	0.174
Creatinine (μmol/L) (*n* = 122)	76.0 ± 14.9	76.6 ± 12.6	82.1 ± 24.6	0.501
Uric acid (μmol/L) (*n* = 122)	41.8 ± 90.1	313.1 ± 86.9	336.8 ± 82.4	0.742
Glomerular filtration rate, mL/min/1.73 m^2^ (*n* = 122)	87.8 ± 13.7	86.0 ± 10.8	81.7 ± 18.9	0.448
*Gleason score (n = 143)*				
6	6 (5.6)	1 (5.3)	0 (0)	0.880
7	29 (26.9)	7 (36.8)	3 (18.8)	
8	29 (26.9)	4 (21.1)	3 (18.8)	
9	35 (32.4)	6 (31.6)	8 (50.0)	
10	9 (8.3)	1 (5.3)	2 (12.5)	
*Metastases (n = 182)*				
Yes	138 (63.6)	22 (59.5)	22 (73.3)	0.477
No	79 (36.4)	15 (40.5)	8 (26.7)	
*Type of therapy (n = 284)*				
Initial diagnosis	75 (34.6)	10 (27.0)	8 (26.7)	0.654
Radical prostatectomy	52 (24.0)	6 (16.2)	9 (30.0)	
ADT (with or without chemotherapy)	46 (21.2)	11 (29.7)	6 (20.0)	
Radical prostatectomy with other treatments	33 (15.2)	8 (21.6)	6 (20.0)	
Radiotherapy (with or without chemotherapy)	7 (3.2)	0 (0)	1 (3.3)	
Chemotherapy	2 (0.9)	1 (2.7)	0 (0)	
Targeted therapy	2 (0.9)	1 (2.7)	0 (0)	

TPSA, total prostate-specific antigen; ALT, alanine transaminase; AST, aspartate transaminase. ADT, androgen deprivation therapy; SUV_max_, maximum standardized uptake value; SUV_mean_, mean standardized uptake value.

### 3.3. Imaging characteristics

The imaging characteristics of patients with high, middle and low uptake of the bladder on ^18^F-PSMA-1007 PET/CT are shown in Table 3. ^18^F-PSMA-1007 injected activity was 8.7 ± 1.0 mCi in the high uptake group and 8.8 ± 1.1 mCi in the low uptake group. There were no significant differences in the injected activity between the three groups (*p* = 0.398). The ^18^F-PSMA-1007 injection-to-scan intervals were 182.8 ± 20.2 min, 182.4 ± 13.6 min, and 183.5 ± 16.0 min in the low, middle, and high uptake groups, respectively, which were also not significantly different (*p* = 0.901). In the ^18^F-PSMA-1007 PET/CT imaging, with the obturator internus as a background, there were no differences in the SUV_max_ (*p* = 0.217) and SUV_mean_ (*p* = 0.127) between the three groups. In addition, there were no significant differences in SUV_max_ and SUV_mean_ between the three groups for physiological uptake of parotid gland, kidney, liver, spleen, intestine, and pathological uptake of prostate lesions and metastases.

**Table 3:** Image characteristics stratified by SUV_max_ of the bladder.

Image characteristics	SUV_max_ ≤5	SUV_max_ 5–7.5	SUV_max_ >7.5	*p*
^18^F-PSMA-1007 injected activity (mCi)	8.8 ± 1.1	8.7 ± 1.1	8.7 ± 1.0	0.398
Injection-to-scan interval (min)	182.8 ± 20.2	182.4 ± 13.6	183.5 ± 16.0	0.901
*Bladder*				
SUV_max_	2.2 ± 1.1	6.2 ± 0.7	11.1 ± 3.5	<0.001
SUV_mean_	1.6 ± 0.8	4.9 ± 1.0	8.7 ± 3.1	<0.001
*Prostate (n = 181)*				
SUV_max_	24.0 ± 20.7	25.2 ± 16.2	39.0 ± 46.1	0.301
SUV_mean_	14.3 ± 11.9	15.4 ± 9.8	21.8 ± 23.8	0.256
*Metastatic lesions (n = 182)*				
SUV_max_	20.6 ± 18.8	26.3 ± 44.5	30.4 ± 29.3	0.356
SUV_mean_	13.0 ± 11.8	17.6 ± 30.9	20.1 ± 22.2	0.333
*Parotid gland (n = 282)*				
SUV_max_	27.6 ± 8.4	26.0 ± 9.6	29.5 ± 8.3	0.337
SUV_mean_	18.9 ± 5.6	17.5 ± 6.7	19.9 ± 5.5	0.335
*Liver*				
SUV_max_	14.2 ± 3.8	13.7 ± 4.3	13.1 ± 3.5	0.337
SUV_mean_	10.9 ± 2.9	10.2 ± 3.1	9.8 ± 2.8	0.082
*Kidney*				
SUV_max_	29.2 ± 8.2	29.2 ± 9.3	30.4 ± 8.9	0.659
SUV_mean_	20.9 ± 14.9	20.5 ± 6.8	21.1 ± 6.2	0.549
*Spleen (n = 283)*				
SUV_max_	14.9 ± 4.8	14.2 ± 5.0	15.0 ± 5.3	0.888
SUV_mean_	12.4 ± 4.2	11.7 ± 4.4	12.5 ± 4.7	0.810
*Intestine*				
SUV_max_	12.0 ± 4.8	13.4 ± 5.4	13.9 ± 5.2	0.059
SUV_mean_	8.0 ± 3.2	8.7 ± 3.2	9.1 ± 3.4	0.125
*Obturator internus*				
SUV_max_	0.7 ± 0.2	0.7 ± 0.2	0.8 ± 0.2	0.217
SUV_mean_	0.5 ± 0.1	0.5 ± 0.1	0.6 ± 0.1	0.127

PSMA, prostate-specific membrane antigen; SUV_max_, maximum standardized uptake value; SUV_mean_, mean standardized uptake value.

**Figure 2:**
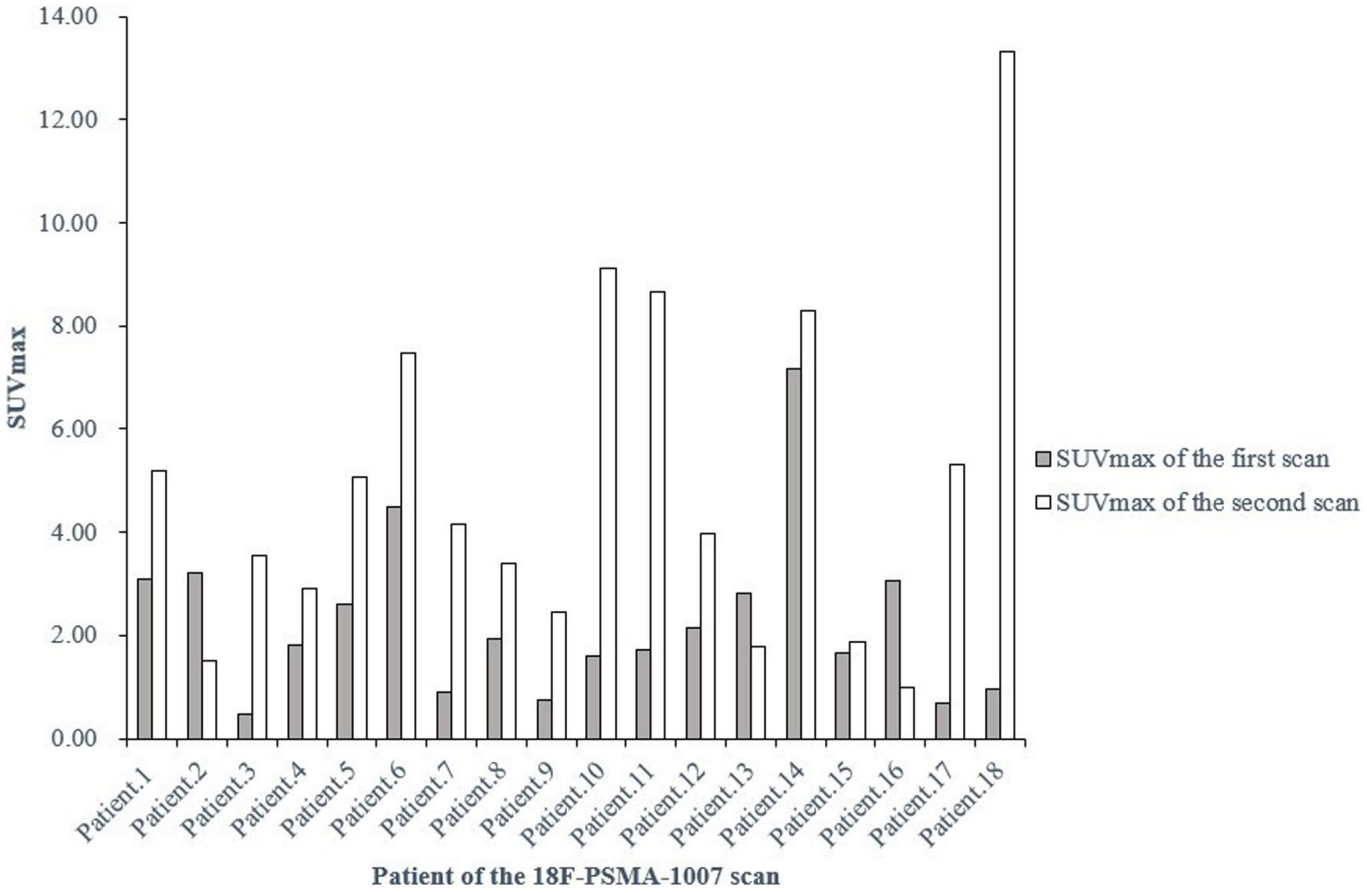
SUV_max_ of the bladder at two ^18^F-PSMA-1007 PET/CT examinations in the same patient at different times. SUV_max_, maximum standardized uptake value.

Additionally, 18 patients in the entire study cohort underwent two or more ^18^F-PSMA-1007 PET/CT scans, and 7 (38.9%) of them presented with different levels of urine ^18^F-PSMA-1007 uptake between scans (Figure 2). Whereas, there was no correlation between the above-mentioned patients who underwent 2 PET/CT scans based on their clinical characteristics (laboratory examinations, treatment modalities, etc.) and imaging characteristics (injected activity, injection-to-scan interval).

## 4. Discussion

^18^F-PSMA-1007 is a new PSMA-based radiopharmaceutical that can replace the routinely used radiotracer ^68^Ga-PSMA-11 for the evaluation of PCa patients. A retrospective study by Giesel et al. (20) showed that ^18^F-PSMA-1007 had a higher detection rate of biochemical recurrence than ^68^Ga-PSMA-11, even in patients with low PSA levels (≤0.5 ng/mL) in patients. In addition, ^18^F-PSMA-1007 also showed higher sensitivity to low-grade lesions (21). These findings suggest that ^18^F-PSMA-1007 may have a significant impact on the further management of the disease. ^18^F-PSMA-1007 is mainly cleared by the liver and bile and therefore urinary radioactive interference is substantially reduced, thus allowing a better assessment of lesions in the prostate and pelvic regions, making its use in clinical practice increasingly widespread. However, we observed in our clinic that 23.6% of patients had SUV_max_ >5 in the bladder, and of these, 10.6% had SUV_max_ >7.5 in the bladder, which could potentially affect the assessment of lesions in the peri-bladder region. Therefore, to determine which factors may lead to the currently unidentified increase in urinary uptake, this study evaluated the clinical characteristics and imaging characteristics of patients who underwent ^18^F-PSMA-1007 PET/CT scans.

Firstly, we found that the increase in urine uptake was not related to individual characteristics of patients such as age, height, weight, and Gleason score after excluding the effect from the background distribution by comparing the ^18^F-PSMA-1007 uptake in the obturator internus. In addition to this, ^18^F-PSMA-1007 is mainly metabolized by the liver and temporarily stored in the renal parenchyma, but endocrine therapy, chemotherapy and other drugs in prostate cancer patients may cause injury to liver and kidney function. It has also been shown that renal ^18^F-PSMA-1007 uptake parameters correlate with EGFR and can indicate renal cortical function (21). However, high ^18^F-PSMA-1007 uptake in the bladder cannot be linked to liver and kidney function, as no significant differences were found by analyzing different treatment methods as well as liver and kidney function indicators of the patients. Moreover, in patients with high tumor burden, ^68^Ga-PSMA-11 exhibits a tumor sink effect, i.e., it decreases the distribution of radioactivity in normal organs (kidney, liver, spleen, etc.) (22). However, we found no correlation between bladder ^18^F-PSMA-1007 distribution and physiological distribution in normal organs or the presence or absence of metastases. However, this relationship is not clear and needs to be further confirmed by measuring the specific tumor burden in a prospective study.

Despite the high stability of the ^18^F-PSMA-1007 tracer (23), free ^18^F, a higher proportion of unlabeled material or a smaller molar activity may limit the distribution of this tracer. Our study showed that the injected activity and the interval between injection and examination did not correlate with bladder ^18^F-PSMA-1007 distribution. Relt et al. (24) also indicated that ^18^F-PSMA-1007 post-injection time was only weakly correlated with liver uptake, both the time after drug synthesis and the time after injection were not related to physiological uptake in other organs or Pathological uptake. Giesel et al. (14) also showed that there were no differences by comparing the bladder ^18^F-PSMA-1007 distribution at 1 h and 3 h in 10 patients, but the mean SUV_max_ value of 5 in the bladder in their study was higher than that of 3.6 in our study, which may be related to the small number of cases they included. Of course, it would be more convincing if urine samples were tested after injection and examination, but this needs to be verified in a prospective study. In addition to this, we also compared the bladder ^18^F-PSMA-1007 distribution between the two examinations in the same patient and found that some patients showed a significant change in urine ^18^F-PSMA-1007 distribution, which is more likely to affect our accurate assessment of the patient’s disease.

Furthermore, although PSMA is highly overexpressed in most prostate cancers, it has been shown that this correlation is not perfect and that tracer uptake may also be present in tissues with low PSMA expression or negative PSMA expression (25). Therefore, incidental uptake could also be due to non-specific uptake of ^18^F-PSMA-1007 on still-unknown targets.

This study has certain limitations: first, this study is retrospective and there are limitations in data collection. Second, the sample volume was small, and a multicenter, large sample study could be conducted subsequently. And finally, it is unknown whether ^18^F-PSMA-1007 will heterodimerize over time, which can be subsequently confirmed by urine sample assay in prospective experiments.

## 5. Conclusion

In ^18^F-PSMA-1007 PET/CT imaging, there was a certain amount of ^18^F-PSMA-1007 distribution in the bladder, and in some patients, the bladder distribution was close to the prostate lesion, which may affect the observation of peri-prostate lesions. However, this study showed that there were no correlations with age, height, weight, Gleason score, metastases, different treatment methods, liver and kidney function levels, TPSA levels, ^18^F-PSMA-1007 injected activity, the interval from injection to scan, the physiological distribution of parotid gland, kidney, liver, spleen, intestine, obturator internus, and the pathological distribution of prostate lesions, metastatic lesions in the distribution of ^18^F-PSMA-1007 in the bladder. Therefore, more research is still needed to find the causes of this problem, so as to improve the disease assessment in this type of prostate cancer patients.

## Data availability statement

The raw data supporting the conclusions of this article will be made available by the authors, without undue reservation.

## Ethics statement

The study was ethically approved by the Institutional Ethics Committee (Ethics Committee of Sichuan Cancer Hospital, JS-2017-01-02). Informed consent was obtained from all the subjects involved in the study.

## Author contributions

JD, YY, XT, and YL designed the project. JD and XT wrote the manuscript. ZY, YZ, and SQ organized data. JD, SC, and MZ analyzed data. YY, YK, HL, and ZC reviewed the data and the manuscript. All authors contributed to the article and approved the submitted version.

## Funding

This study was supported by funds from Science & Technology Department of Sichuan Province (No. 22ZDYF1359), Sichuan Medical Health and Health Care Promotion Institute (KY2022SJ0260) and Sichuan Cancer Hospital Outstanding Youth Funding (YB 2023022).

## Conflict of interest

The authors declare that the research was conducted in the absence of any commercial or financial relationships that could be construed as a potential conflict of interest.

## Publisher’s note

All claims expressed in this article are solely those of the authors and do not necessarily represent those of their affiliated organizations, or those of the publisher, the editors and the reviewers. Any product that may be evaluated in this article, or claim that may be made by its manufacturer, is not guaranteed or endorsed by the publisher.
